# Supplementary material for: Local orbital degeneracy lifting as a precursor to an orbital-selective Peierls transition
Source: Nat Commun. 2019 Aug 13;10:3638. doi: 10.1038/s41467-019-11372-w (PMC6692321; doi:10.1038/s41467-019-11372-w)
Supplement: Supplementary file 1 — Supplementary Information [file 41467_2019_11372_MOESM1_ESM.pdf]

Local orbital degeneracy lifting as a precursor to an orbital-selective Peierls transition - Supplementary Information-

E. S. Bozin et al.

(Dated: June 28, 2019)

Supplementary Note 1: Impact of electron doping

Here we consider in greater detail the general effects of the electron doping achieved by Zn-substitution as shown in Fig. 3(c) of the Main Text. In this Figure, the PDF of the low-zinc content sample ($x = 0$ in $(Cu_{1-x}Zn_x)Ir_2S_4$) is shown in blue and that of the heavily substituted sample ($x = 0.7$) is in red. We see a number of effects. First, in the higher-r region, it is evident that the peaks are shifting to the right with increasing doping and that the lattice parameter is expanding [1] (all data were collected at room temperature) (See also Supplementary Fig. 1 and related discussion of the Fd$\bar{3}$m model parameters). This is unlikely to be a steric effect because Zn^{2+} has the same size as Cu^{1+} that it replaces, 60 pm [2]. It is therefore evidence that the chemical substitution of Cu by Zn causes electronic doping in the Ir sublattice. A second observation is that the PDF peaks sharpen with increased Zn content. This is the opposite of what is expected on steric grounds because any small difference in the size of Zn and Cu will lead to structural relaxation to accommodate different sized dopant ions, leading to a peak broadening.

As was discussed in the Main Text in relation to the MI transition, PDF peaks can sharpen if the overall symmetry of the structure increases, even as other effects such as thermal motion or steric structural relaxations are tending to broaden the peaks. The peak sharpening is therefore strong evidence that the local tetragonal distortion is being removed by the zinc substitution. This view is confirmed by the fit of the cubic model to the Zn 70% content experimental PDF in Fig. 3(d). The cubic model fits well over the entire r-range, showing that the local structural distortion has largely disappeared by 70% Zn content, at which point the nominal electron count in the t_{2g} states of the Ir should be 5.85. As the electron count approaches 6 the driving force for a JT distortion diminishes and approaches zero, and so the experimental observations support the view that the distortion has a Jahn-Teller origin.

Increased zinc doping causes intensity to transfer from the lower-r to the higher-r side of the 3.5 Å peak. This is evident in Fig. 3(c), where the difference curve $\Delta G(r) = G_{x=0}(r) - G_{x=x}(r)$. As we have discussed above, the zinc substitution adds electrons to nearly filled t_{2g} manifold of the Ir, which will have the effect of populating the anti-bonding molecular orbitals between neighboring Ir ions and increasing their average separation, as observed. If there is no orbital degeneracy lifting and the charge were evenly distributed among all of the t_{2g} orbitals, this would simply increase the Ir-Ir distance in the cubic model, r_c. An increase in r_c, is indeed observed with increased Zn content. However, a second effect is also evident, which is a change in the tetragonality of the local structure that results in long and short Ir-Ir bonds

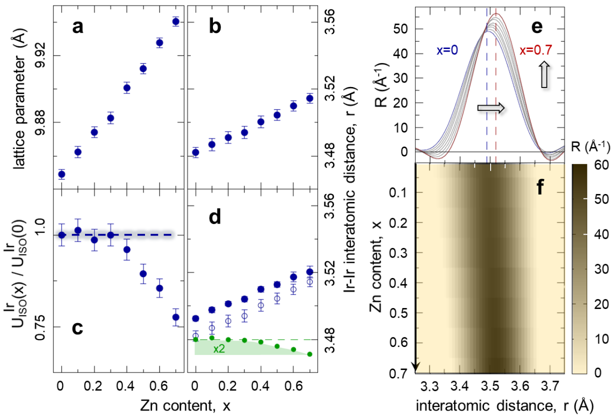

Supplementary Figure 1. **Considerations of $(Cu_{1-x}Zn_x)Ir_2S_4$ at 300 K.** **a** Cubic Fd$\bar{3}$m lattice parameter. **b** Ir-Ir distance obtained from cubic Fd$\bar{3}$m model. **c** Relative isotropic atomic displacement parameter (ADP) of Ir. Horizontal dashed line provides $CuIr_2S_4$ reference. **d** Centroid of the nearest neighbor Ir-Ir PDF peak (solid blue symbols). Average cubic distance shown in **b** is shown by open blue symbols as a reference. Solid green symbols show the difference between the two, multiplied by a factor of 2 for clarity. Error bars represent estimated standard uncertainties on the refined parameters. **e** Stack of the PDF data, shown in form of radial distribution function R(r), [3] over a narrow r range featuring the Ir-Ir nearest neighbor peak. Solid blue profile corresponds to $CuIr_2S_4$, solid red profile corresponds to 70 % Zn substitution. Profiles corresponding to other compositions, in 10 % Zn increments, are shown as solid gray lines. Vertical dashed lines mark the peak centroid of the extreme compositions. Arrows indicate trends. **f** False color intensity representation of the data shown in panel **e**.

(Δr_t). This information is summarized in Fig. 3(i) of the Main Text which plots Δr_t vs. r_c for both the Zn substitution and Cr substitution series. Δr_t is a measure of the orbital degeneracy lifting and the partition of charge between completely and partially filled orbitals, whereas r_c depends on both the average charge filling in the t_{2g} manifold and any steric effects. If in Fig. 3(i) we assume that Δr_t vs. r_c is linear for the Zn substitution series, we can extrapolate to $x = 1$ and we see that the extrapolated tetragonal distortion becomes zero, as expected (filled t_{2g} manifold). Conversely, Cr substitution, which is increasing from right to left in Fig. 3(i), decreases the average Ir-Ir distance whilst increasing the tetragonality, again consistent with the notion that the tetragonal distortion is stabilized by compressive strain.

Supplementary Note 2: Notes on fluctuating ODL state

The state suggested by the experimental evidence is one in which selected partially filled bonding orbitals fluctuate between Ir-Ir pairs. We therefore refer to this state

as a fluctuating ODL state. While the xPDF technique, based on total scattering data, is sensitive to the presence of local distortions and cannot discern whether these are static or dynamic, we expect the high temperature ODL state to be dynamic. This state is similar to the resonating valence bond state that Anderson proposed [4] to explain the low-temperature physics of the cuprates, with the important distinction that the fluctations are here thermally excited and not quantum in nature. This might imply electron dynamics that are hopping in nature, which was indeed observed [5]. Interestingly, at low temperature, $CuIr_2Se_4$ does not form the charge ordered dimer phase and it has no MI transition, as mentioned in the Main Text. This implies that the ODL state is an important precursor to the low-temperature insulating dimer state, and the absence of the ODL state in $CuIr_2Se_4$ inhibits the formation of the charge ordered insulating state at low temperature.

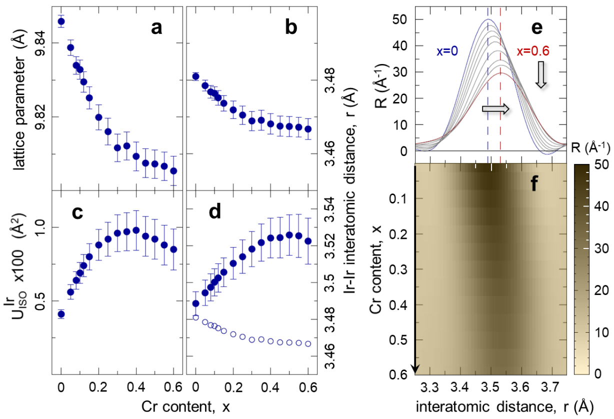

Supplementary Figure 2. **Considerations of $Cu(Ir_{1-x}Cr_x)_2S_4$ at 300 K.** **a** Cubic Fd$\bar{3}$m lattice parameter. **b** Ir-Ir distance obtained from cubic Fd$\bar{3}$m model. **c** Isotropic atomic displacement parameter (ADP) of Ir. **d** Centroid of the nearest neighbor Ir-Ir PDF peak (solid blue symbols). Average cubic distance shown in **b** is shown by open blue symbols as a reference. Error bars represent estimated standard uncertainties on the refined parameters. **e** Stack of the PDF data, shown in form of radial distribution function $R(r)$, [3] over a narrow r range featuring the Ir-Ir nearest neighbor peak. Solid blue profile corresponds to $CuIr_2S_4$, solid red profile corresponds to 60 % Cr substitution. Profiles corresponding to other Cr compositions, in variable increments, [6] are shown as solid gray lines. Vertical dashed lines mark the peak centroid of the extreme compositions. Arrows indicate trends. **f** False color intensity representation of the data shown in panel **e**.

Supplementary Note 3: Phenomenology of the Ir-Ir PDF peak in Zn and Cr substituted $CuIr_2S_4$ variants

The PDF observations at 300 K in $(Cu_{1-x}Zn_x)Ir_2S_4$ and $Cu(Ir_{1-x}Cr_x)_2S_4$ are summarized in Supplementary Fig. 1 and Supplementary Fig. 2. $(Cu_{1-x}Zn_x)Ir_2S_4$ data in $0 \leq x \leq 0.7$ range of Zn concentrations and $Cu(Ir_{1-x}Cr_x)_2S_4$ data for $0 \leq x \leq 0.6$ were modeled using cubic Fd$\bar{3}$m model over a broad r-range $10 \leq r \leq 50$ Å. Here we focus on both explicitly refined as well as derived quantities and their evolution with the substituent content.

In $(Cu_{1-x}Zn_x)Ir_2S_4$ the lattice parameter and derived cubic Ir-Ir nearest neighbor distance both increase with Zn content, as seen in Supplementary Fig. 1(a),(b). This effect is predominantly electronic in character, as discussed. On the other hand, isotropic atomic displacement parameter, U_{ISO}, of Ir, shown normalized to its $x = 0$ value in Supplementary Fig. 1(c), reduces with increased Zn content. This implies that the dominant component of disorder, associated with the ODL state, is being removed from the system. Interestingly, this trend appears to be more rapid for compositions greater than $x = 0.3$ for which superconductivity is observed, [1] as indicated by a rapid departure of this parameter from the reference dashed horizontal line. Simultaneously, the average and local values of the nearest neighbor Ir-Ir separation coalesce, Supplementary Fig. 1(d), confirming that the local distortion is being removed. This trend can also be seen directly in the PDF data, Supplementary Fig. 1(e),(f). There, PDF is shown as radial distribution function (RDF), R(r). [3] One readily sees that there is a transfer of intensity from the leading to the trailing edge of this peak. However, while the leading edge shifts to the right, the trailing edge does not. While this results in a slight shift of the apparent peak centroid towards higher distances, the main effect is that the absolute peak height increases and the peak width narrows. This in turn implies narrowing of the underlying Ir-Ir distance distribution, consistent with the increase in the local symmetry as the local ODL state is being destabilized, as discussed. Notably, while Zn substitution introduces one electron per Zn, this charge difference is too small to affect the PDF peak intensities through a change of the x-ray scattering contrast, and the observed change in the PDF peak intensity comes exclusively from the redistribution of nearest neighbor Ir-Ir interatomic distances.

In $Cu(Ir_{1-x}Cr_x)_2S_4$ the lattice parameter and derived cubic Ir-Ir nearest neighbor distance both decrease with Cr content, as seen in Supplementary Fig. 2(a),(b). This is not surprising, given that the ionic radii of both Cr^{3+} and Cr^{4+} are smaller than these of Ir^{3+} and Ir^{4+} in octahedral coordination [2], so this substitution results in overall reduction in the unit cell volume, as reported. [7] Notably in this case, isotropic atomic displacement parameter, U_{ISO}, of Ir, Supplementary Fig. 2(c), increases with increasing Cr content. This indicates that the overall disorder, both quenched component associated with the Cr/Ir size mismatch and that associated with the ODL state, increases. This trend is initially dramatic, but appears to slow down around $x = 0.5$ Cr composi-

tion, where the character of substitution is expected to change from Cr^{3+} to Cr^{4+} [6]. Once Cr is introduced to the system, the average and local values of the nearest neighbor Ir-Ir separation start diverging away from each other, Supplementary Fig. 2(d), implying growth of the local distortions upon introduction of compressive chemical pressure which stabilizes the ODL state. This trend can also be seen directly in the RDF data, Supplementary Fig. 2(e),(f). In this case there is an additional change in RDF peak intensity originating from removing nearly 70% of the x-ray scattering electron density from the affected sites where Ir (Z=77) is replaced by Cr (Z=24). We note that the local effects in $Cu(Ir_{1-x}Cr_x)_2S_4$ are highly nontrivial due to a complex convolution of steric, orbital, charge, and spin aspects, implying that there may be multiple sources of local distortions, particularly at higher Cr-content. Further complication stems from the prospect of modified character of the spin-orbit coupling, given that $5d$ Ir is being replaced with $3d$ Cr and randomization of other relevant interactions. Disentangling these complexities could be a natural extension of this work in the future.

SUPPLEMENTARY REFERENCES

[1] Cao, G. *et al.* Suppression of metal-to-insulator transition and appearance of superconductivity in $Cu_{1-x}Zn_xIr_2S_4$. *Phys. Rev. B* **64**, 214514 (2001).

[2] Shannon, R. D. Revised effective ionic radii and systematic studies of interatomic potentials. *Acta Crystallogr. A* **32**, 751,767 (1976).

[3] Egami, T. & Billinge, S. J. L. *Underneath the Bragg peaks: structural analysis of complex materials* (Elsevier, Amsterdam, 2012), 2nd edn.

[4] Anderson, P. W. The resonating valence bond state in La_2CuO_4 and superconductivity. *Science* **235**, 1196 (1987).

[5] Takubo, K., Mizokawa, T., Matsumoto, N. & Nagata, S. In-gap state and effect of light illumination in $CuIr_2S_4$ probed by photoemission spectroscopy. *Phys. Rev. B* **78**, 245117 (2008).

[6] Božin, E. S. *et al.* $Cu(Ir_{1-x}Cr_x)_2S_4$: a model system for studying nanoscale phase coexistence at the metal-insulator transition. *Sci. Rep.* **4**, 4081 (2014).

[7] Endoh, R., Awaka, J. & Nagata, S. Ferromagnetism and the metal-insulator transition in the thiospinel $Cu(Ir_{1-x}Cr_x)_2S_4$. *Phys. Rev. B* **68**, 115106 (2003).
